# Complete genome sequence and potential pathogenic assessment of *Flavobacterium plurextorum* RSG‐18 isolated from the gut of Schlegel's black rockfish, *Sebastes schlegelii*

**DOI:** 10.1111/1758-2229.13226

**Published:** 2024-01-31

**Authors:** Jisol Lee, In‐Tae Cha, Ki‐Eun Lee, Youn Kyoung Son, Seoae Cho, Donghyeok Seol

**Affiliations:** ^1^ Department of Agricultural Biotechnology and Research Institute of Agriculture and Life Sciences Seoul National University Seoul South Korea; ^2^ Microorganism Resources Division National Institute of Biological Resources Incheon South Korea; ^3^ eGnome, Inc. Seoul South Korea; ^4^ Department of Surgery Seoul National University Bundang Hospital Seongnam South Korea

## Abstract

*Flavobacterium plurextorum* is a potential fish pathogen of interest, previously isolated from diseased rainbow trout (*Oncorhynchus mykiss*) and oomycete‐infected chum salmon (*Oncorhynchus keta*) eggs. We report here the first complete genome sequence of *F. plurextorum* RSG‐18 isolated from the gut of Schlegel's black rockfish (*Sebastes schlegelii*). The genome of RSG‐18 consists of a circular chromosome of 5,610,911 bp with a 33.57% GC content, containing 4858 protein‐coding genes, 18 rRNAs, 63 tRNAs and 1 tmRNA. A comparative analysis was conducted on 11 *Flavobacterium* species previously reported as pathogens or isolated from diseased fish to confirm the potential pathogenicity of RSG‐18. In the SEED classification, RSG‐18 was found to have 36 genes categorized in ‘Virulence, Disease and Defense’. Across all *Flavobacterium* species, a total of 16 antibiotic resistance genes and 61 putative virulence factors were identified. All species had at least one phage region and type I, III and IX secretion systems. In pan‐genomic analysis, core genes consist of genes linked to phages, integrases and matrix‐tolerated elements associated with pathology. The complete genome sequence of *F. plurextorum* RSG‐18 will serve as a foundation for future research, enhancing our understanding of *Flavobacterium* pathogenicity in fish and contributing to the development of effective prevention strategies.

## Graphical abstract

The complete genome was constructed to investigate the genetic potential of RSG‐18 for pathogenicity. Comparative genome analysis with other well‐known flavobacterial fish pathogens revealed that RSG‐18 possesses putative virulence genes associated with protein degradation and resistance to fish phagocytes, as well as various types of secretion systems essential for infection.

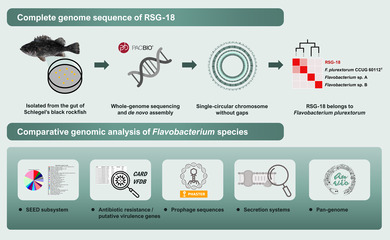

## INTRODUCTION

The genus *Flavobacterium* has been found in various habitats, such as rivers (Sheu et al., 2016), lakes (Kim et al., 2009), tidal flats (Bae et al., 2018), rhizosphere (Madhaiyan et al., 2010) and wetlands (Phurbu et al., 2019). Several *Flavobacterium* spp. have mutualistic interactions with plants by producing phytohormones or fixing nitrogen (Kolton, 2016). However, other *Flavobacterium* spp. such as *Flavobacterium columnare*, *Flavobacterium psychrophilum* and *Flavobacterium branchiophilum* have been reported as pathogenic to fish. Flavobacterial disease impacts farmed and wild fish alike, causing severe economic losses in the fishing industry (Ostland et al., 1994; Starliper, 2011; Thomas‐Jinu & Goodwin, 2004). In addition, *Flavobacterium plurextorum*, *Flavobacterium araucananum* and *Flavobacterium piscis* have been isolated from diseased fish (Kämpfer et al., 2012; Zamora et al., 2013; Zamora et al., 2014), which have not been studied much yet, but are thought to have potential pathogenicity (Kumru et al., 2020; Loch & Faisal, 2015). Among them, *F. plurextorum* was first isolated from diseased rainbow trout (*Oncorhynchus mykiss*) in 2008 (Zamora et al., 2013). Large‐scale comparative genomic analysis has revealed that *F. plurextorum* has partial type IV secretion system (T4SS) and type VI secretion system subtype 3 (T6SS^iii^) which can contribute to pathogenicity (Kumru et al., 2020). Recently, *F. plurextorum* was also isolated from oomycete‐infected chum salmon (*Oncorhynchus keta*) eggs, showing pathogenic potential to fish eggs (Sakaguchi et al., 2022).

Here, we present the first complete genome of the *F. plurextorum* species isolated from the gut of Schlegel's black rockfish, *Sebastes schlegelii*. To investigate any pathogenic potential, comparative genome analysis with previously reported *F. plurextorum* genomes and other major flavobacterial fish pathogens was conducted. The genetic characteristics of *F. plurextorum* revealed in this study will broaden our understanding of the relationship between fish and *Flavobacterium*.

## RESULTS AND DISCUSSION

### Genome characteristics

Strain RSG‐18 was isolated from the gut of Schlegel's black rockfish, *S. schlegelii*. The gut samples were serially diluted by R2A broth. An aliquot of 200 μL was spread onto R2A agar at 20°C for 3 days. After 3 days, yellow, rough and irregular colonies were observed (Figure S1). Genomic DNA was extracted using a genomic DNA extraction kit (RBC Bioscience©) following the manufacturer's bacterial protocol. Library preparation was performed using the SMRTbell® Template Preparation Kit (v.1.0), followed by sequencing on the PacBio RS II platform (Pacific Biosciences©).

All bioinformatics tools used in this study are summarized in Table S1. De novo assembly was conducted by Flye (v.2.8.3) with asm‐coverage 100 options, followed by polishing using GCpp (v.2.0.2) to further correct errors in the assembled genome. The polished genome was adjusted to the starting coordinate position (*dnaA* gene) and rotated using the fixstart function in Circlator (v.1.5.5). Assessment was performed by BUSCO (v.5.2.2) with flavobacteriales_odb10 dataset.

**FIGURE 1:**
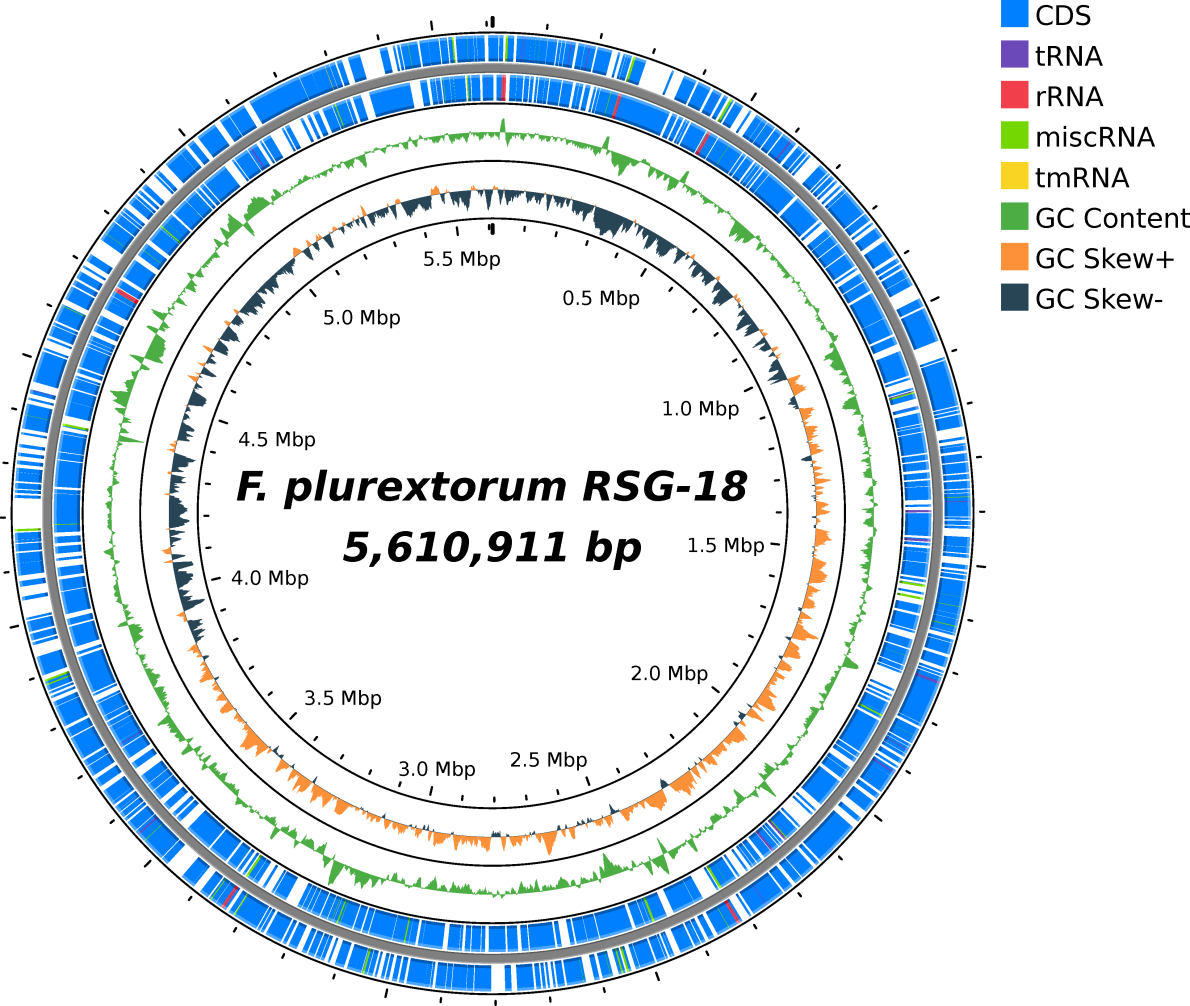
Complete genome map of RSG‐18. From outside to the centre: CDS (blue), tRNA (purple), rRNA (red), miscRNA (yellow‐green), tmRNA (yellow), GC content (green), GC Skew+ (orange), GC Skew– (navy). The map was generated using Proksee (https://proksee.ca/).

As a result of de novo assembly and polishing, a single circular chromosome without gaps was produced. The complete genome sequence of RSG‐18 was covered to a depth of 763 x with a total size of 5,610,911 bp and a GC content of 33.57%. Assembly completeness evaluated with BUSCO indicated 100% completeness and duplication rate of 1.2% using the flavobacteriales lineages. Genome annotation by Prokka (v.1.14.6) yielded 4858 coding sequences (CDS), 18 rRNAs, 63 tRNAs and 1 tmRNA (Table 1, Figure 1). Web‐based annotation tools eggNOG‐mapper (v.2.1.6) (http://eggnog-mapper.embl.de/) was performed for additional annotation of the putative function of the genes based on orthologs. The largest protein‐coding category (except ‘Function unknown’) in RSG‐18 was ‘Cell wall/membrane/envelope biogenesis (M)’ (Table S2).

**TABLE 1:** General features of *Flavobacterium plurextorum* RSG‐18 and the minimum information about a genome sequence (MIGS) mandatory information.

Item	Description
*General features*	
Classification	Domain Bacteria
	Phylum Bacteroidota
	Class Flavobacteriia
	Order Flavobacteriales
	Family *Flavobacteriaceae*
	Genus *Flavobacterium*
	Species *Flavobacterium plurextorum*
	Strain RSG‐18
Gram stain	Negative
Cell shape	Rod
Pigmentation	Yellow
Optimal temperature	20–25°C
*MIGS data*	
Investigation type	Bacteria
Submitted to INSDC	CP100442 (GenBank)
BioProject accession	PRJNA855323
BioSample accession	SAMN29489144
Project name	Complete genome sequence of *Flavobacterium plurextorum* RSG‐18
Geographic location	Republic of Korea
Collection date	8 January 2013
*Sequencing*	
Assembly method	Flye v.2.8.3
Genome representation	Full
Genome coverage	763.0x
Sequencing technology	PacBio RS II
*Genome attribute*	
Genome size (bp)	5,610,911
Contig GC content (%)	33.57
Total genes	5001
Protein coding genes	4858
tRNA genes	63
rRNA genes	18
tmRNA	1

### Taxonomic status

Barrnap (v.0.9) was used for the extraction of 16S rRNA gene sequences from the assembled genome. The sequences predicted by Barrnap were submitted to the BLASTN (https://blast.ncbi.nlm.nih.gov) and EzBioCloud database (https://www.ezbiocloud.net) to identify phylogenetic neighbours. Subsequently, the 16S rRNA gene sequences of 18 neighbouring *Flavobacterium* strains (14 species), including type strains, and *Myroides guanonis* IM13^T^ were obtained. SINA aligner (v.1.2.11) and trimAl (v.1.4.rev15) were used for multiple sequence alignment and alignment trimming, respectively. The phylogenetic tree was built using the maximum‐likelihood (ML) algorithm in IQ‐TREE (v.2.1.4) with 1000 bootstrap replicates (Minh et al., 2020). The phylogenetic analysis based on the 16S rRNA gene sequence revealed that RSG‐18 was clustered with the species *F. plurextorum* (Figure S2A). The 16S rRNA gene sequence of RSG‐18 was completely identical to *F. plurextorum* CCUG 60112^T^, isolated from Trout eggs (*O. mykiss*) in Spain in 2008 (Table S3) (Zamora et al., 2013). RSG‐18 was isolated from the gut of *S. schlegelii* in 2013, showing the mutational robustness of the 16S rRNA gene despite differences in the time of isolation, fish species, etc. As was the case with *F. psychrophilum* (Apablaza et al., 2013), this occurrence was not uncommon.

Average nucleotide identity (ANI) was calculated using Pyani (v.0.2.11) to determine the taxonomic status based on the whole‐genome sequence. A total of 13 *Flavobacterium* genomes, collected from the NCBI RefSeq database (https://www.ncbi.nlm.nih.gov/refseq/) (Table 2), were subjected to comparison. RSG‐18 exhibited ANI values above 98% with two strains of *F. plurextorum*. Additionally, ANI values were 92.5% with *Flavobacterium oncorhynchi* and between 71.4% and 82.9% with the remaining *Flavobacterium* species (Figure S2B). According to the currently accepted criterion (Figueras et al., 2014), which considers two different strains as the same species when they exhibit an ANI value above 95% ~ 96%, it is established that RSG‐18 belongs to the species *F. plurextorum*. *Flavobacterium plurextorum* CCUG 60112^T^ and *F. plurextorum* 2 exhibit genome sizes of 5.06 and 5.04 Mbp, respectively, so RSG‐18's genome size is about 0.6 Mbp longer. The GC content of all three strains is similar at 33%.

**TABLE 2:** General information on *Flavobacterium* species used in this study.

Taxon name	Strain name	Accession	Source	Host status	Report on pathogenicity
*Flavobacterium plurextorum*	RSG‐18	GCF_024638035	*Sebastes schlegelii*	—	—
*Flavobacterium plurextorum*	2	GCF_002754275^a^	Missing	—	—
*Flavobacterium plurextorum*	CCUG 60112^T^	GCF_002217395	*Oncorhynchus mykiss*	Bacterial septicemia	(Zamora et al., 2013)
*Flavobacterium columnare*	NBRC 100251^T^	GCF_007990835	*Oncorhynchus tshawytscha*	Columnaris disease	(Thomas‐Jinu & Goodwin, 2004)
*Flavobacterium psychrophilum*	ATCC 49418^T^	GCF_000754405	*Oncorhynchus kisutch*	Bacterial coldwater disease	(Starliper, 2011)
*Flavobacterium branchiophilum*	DSM 24789^T^	GCF_006716585	*Oncorhynchus masou*	Salmonid bacterial gill disease	(Good et al., 2015; Ostland et al., 1994)
*Flavobacterium tructae*	ATCC BAA‐2541^T^	GCF_002217445	*Oncorhynchus tshawytscha*	Bacterial gill disease	(Loch & Faisal, 2014)
*Flavobacterium johnsoniae*	UW101^T^	GCF_000016645	Soil	—	(Suebsing & Kim, 2012)
*Flavobacterium succinicans*	DSM 4002^T^	GCF_900114945	Fingerling chinook salmon	Bacterial gill disease	(Good et al., 2015)
*Flavobacterium hydatis*	ATCC 29551^T^	GCF_002217335	Salmon	Bacterial disease	(Mantareva et al., 2022)
*Flavobacterium chilense*	DSM 24724^T^	GCF_900142685	Diseased rainbow trout (*Oncorhynchus mykiss*)	Bacterial gill disease	(Kämpfer et al., 2012)
*Flavobacterium araucananum*	DSM 24704^T^	GCF_003148525	Atlantic salmon (*Salmo salar*)	Bacterial gill disease	(Kämpfer et al., 2012; Loch & Faisal, 2014)
*Flavobacterium oncorhynchi*	CCUG 59446^T^	GCF_002217355	*Oncorhynchus mykiss*	Diseased	(Zamora et al., 2012)

^a^ It was registered as *Flavobacterium* sp. 2 in NCBI, however, the average nucleotide identity (ANI) analysis confirmed that it is belonging to *Flavobacterium plurextorum*.

### Comparative genomic analysis

To predict potential pathogenicity, a comparative analysis was performed using the 10 *Flavobacterium* species reported to cause flavobacterial fish diseases (Table 2) (Loch & Faisal, 2015). We used several functional classification systems with different database sizes, hierarchies and purposes to compare genomic features more comprehensively (Zeller & Huson, 2022).

**FIGURE 2:**
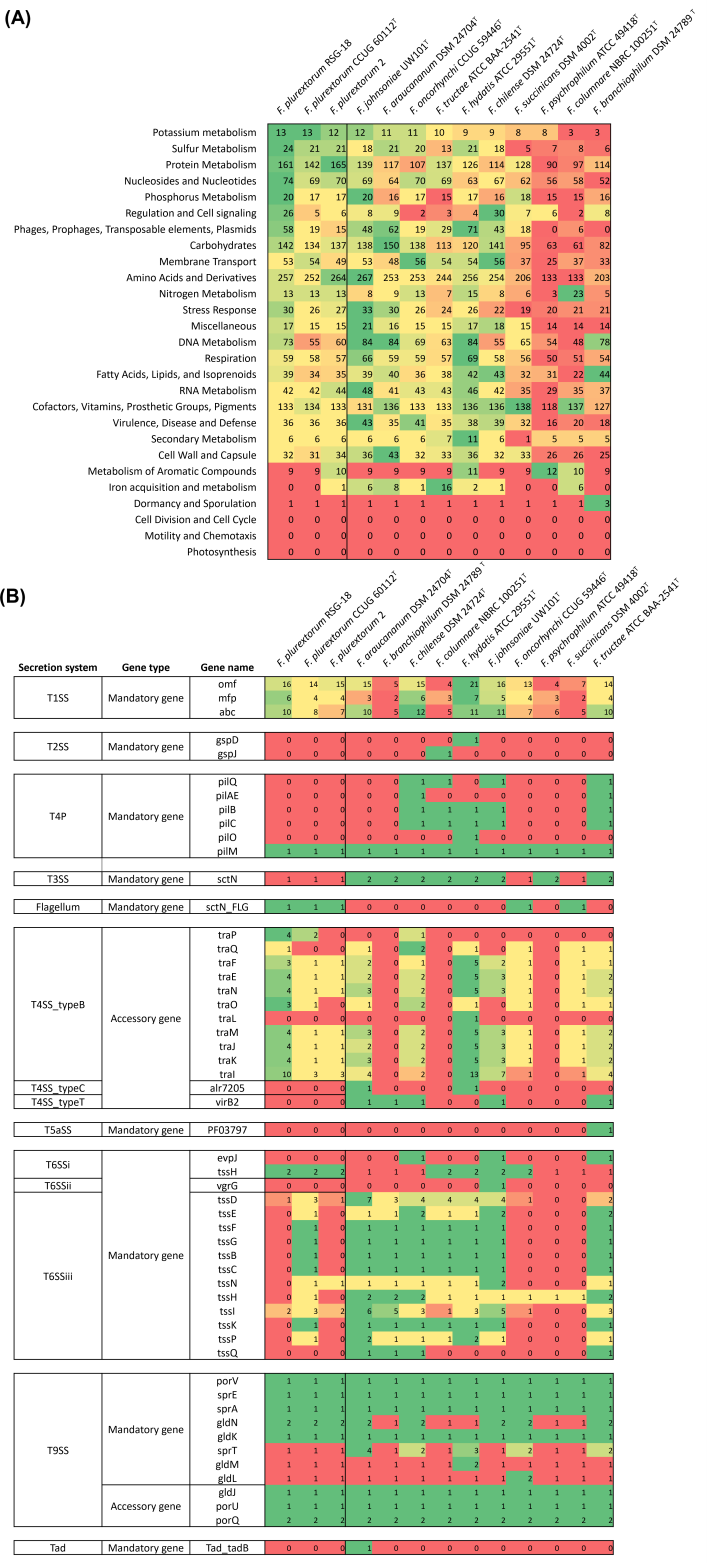
Summary for comparative genomic analysis. (A) The number of genes belonging to 27 RAST SEED subsystem categories for 13 *Flavobacterium* strains is displayed. (B) Secretion systems in *Flavobacterium* species. The number of elements identified in each genome is displayed. In a specific category, a higher gene count is depicted with a brighter green colour, while a lower gene count is represented by a brighter red colour.

Rapid Annotations using Subsystems Technology (RAST) (v.2.0) was used with default parameters to categorize SEED subsystem of *Flavobacterium* genomes (Aziz et al., 2008). In all *Flavobacterium* species, no genes associated with ‘cell division and cell cycle’, ‘motility and chemistry’ and ‘photosynthesis’ were identified. However, genes corresponding to the other 24 SEED subsystems were found in all or most of the species (Figures 2A, S3A,B). Notably, all analysed genomes contained genes classified under the ‘Virulence, Disease and Defense’ category. However, even for obvious pathogens in this category, no genes related to adhesion or toxins were annotated, and most were categorized as genes for resistance to heavy metals or antibiotics (Figure S4). For example, metallo‐β‐lactamase (MBL) fold metallo‐hydrolase, which has catalytic activity for a wide range of β‐lactam antibiotics (Colson et al., 2020), and *gyrA*/*gyrB* associated with quinolone resistance were identified in all genomes. While *gyrA* and *gyrB* typically encode DNA gyrase subunits involved in regulating DNA supercoiling, functional mutations in these genes can confer antibiotic resistance (Mata et al., 2018). However, none of the *Flavobacterium* species used in the comparison, including RSG‐18, exhibited variants known to induce quinolone resistance in these genes (Table S4) (Declercq et al., 2021; Izumi & Aranishi, 2004; Shah et al., 2012).

For the detection of antibiotic resistance genes (ARGs) and putative virulence, ABRicate (v.1.0.1) (https://github.com/tseemann/abricate) was used with the CARD and VFDB databases, respectively (Table S5). A total of 16 ARGs were detected in nine *Flavobacterium* genomes based on the CARD database. The *JOHN‐1* gene was detected in nine genomes, including RSG‐18. *JOHN‐1* is a β‐lactamase that was initially discovered in *Flavobacterium johnsoniae* and has been reported to be resistant to penicillin, cephalosporin and carbapenem antibiotics (Naas et al., 2003).

From the VFDB, a total of 61 putative virulence genes were predicted from 13 *Flavobacterium* genomes. High temperature protein B (*htpB*) gene was identified in all genomes, including RSG‐18. However, it showed less than 70% nucleotide identity, and the actual gene annotated by Prokka was *groL*, which encodes a chaperonin GroEL. According to Valenzuela‐Valderas et al., GroEL in *Escherichia coli* differs by several amino acids from HtpB in *Legionella pneumophila* (the reference species for *htpB* in the VFDB), leading to a functionally different protein folding that may not be conducive to infection (Valenzuela‐Valderas et al., 2022). Therefore, it is not expected to be virulent and has only been reported to be immunogenic in *Flavobacterium* (Liu et al., 2012; Valenzuela‐Valderas et al., 2022).

The putative virulence gene *katA*/*katB* involved in catalase production was present in three species of *F. plurextorum* strains, including RSG‐18, *F. araucananum*, *F. branchiophilum*, *F. chilense*, *F. columnare*, *F. hydatis*, *F. oncorhynchi*, *F. psychrophilum* and *F. succinicans*. Although they showed low similarity again, they were indeed annotated as the *katG* encoding catalase/peroxidase known to play an important role in resisting fish phagocytes (Tekedar et al., 2017; Zhang et al., 2018).

In addition, the ATP‐dependent CLP protease proteolytic subunit, *clpP*, was identified only in the *F. plurextorum* genomes. In certain bacteria, *clpP* is important for the degradation of proteins involved in nutrient deficiency, heat‐stress reaction, stationary phase adaptation, cell cycle progression, cell motility, biofilm formation, nutrition and metabolism (Moreno‐Cinos et al., 2019). Since it has a widespread effect on proteins, *clpP* function plays an important role in infectivity and virulence in a number of bacterial pathogens (Bhandari et al., 2018). In summary, reference‐based analysis shows that *F. plurextorum* has several virulence‐related genes, suggesting that *F. plurextorum* has pathogenic potential like other fish pathogens. However, as in the case of *htpB*, candidates from in silico‐based analyses must be verified through experimental investigation to ensure they are actually expressed in infecting fish.

Prophages have a key role in bacterial pathogenicity. They typically encode virulence genes and make significant contributions to the strains' genetic distinctiveness (Canchaya et al., 2004). To identify prophage sequences within genomes, PHAge Search Tool Enhanced Release (PHASTER) (https://phaster.ca) was used. All 13 *Flavobacterium* genomes submitted to PHASTER server had at least one type of phage regions. A total of 21 incomplete and one questionable prophage were identified. The GC content of phages showed a similar tendency in the range of 29.52%–36.28%, with an exception of phage found in RSG‐18, which had a GC content of 42.77%. RSG‐18 hosted three incomplete prophage sequences which included a 10.1 Kb *Escherichia* phAPEC8 (NCBI accession: NC_020079), an 8.2 Kb *Paenibacillus* Likha (NC_048693), and a 9.9 Kb *Bacillus* vB_BsuM‐Goe3 (NC_048652).

The secretion system plays a crucial role in bacterial growth and diverse cellular processes, primarily transporting of proteins from the cytoplasm to the external environment, including bacteria and eukaryotic cells (Costa et al., 2015). To investigate which secretion systems *Flavobacterium* have, an exploration of 22 models of the protein secretion system was conducted using TXSScan (v.1.0.5), an MacSyFinder‐based detection program. All *Flavobacterium* species contained type I secretion system (T1SS; *omf*, *mfp*, *abc*), type III secretion system (T3SS; *sctN*), and type IX secretion system (T9SS; *porV*, *sprE*, *sprA*, *gldN*, *gldK*, *sprT*, *gldM*, *gldL*) (Figure 2B). This finding is consistent with a previous study by Kumru et al., which reported that all genomes of 86 *Flavobacterium* strains isolated from aquatic hosts, mainly fish, possessed T1SS and T9SS secretion systems (Kumru et al., 2020). Additionally, three strains of *F. plurextorum*, *F. oncorhynchi* and *F. succinicans*, were found to contain flagellum‐related gene (*sctN_FLG*), while most genomes exhibited type IV secretion system (T4SS) accessory genes and type VI secretion system (T6SS) mandatory genes.

**FIGURE 3:**
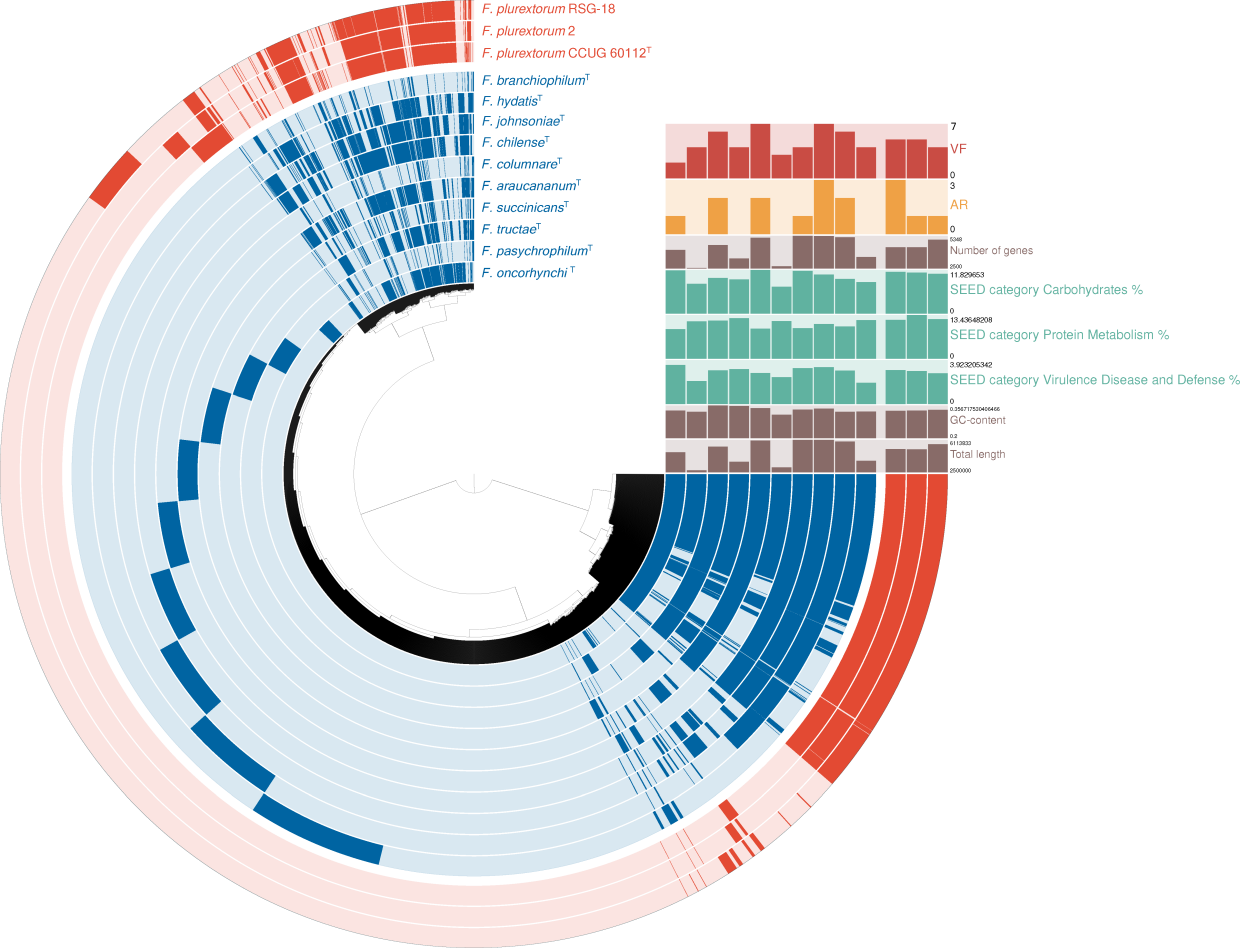
Pangenome of 13 *Flavobacterium* genomes. The outermost red rings represent information from three *Flavobacterium plurextorum* genomes and the blue rings represent 10 *Flavobacterium* genomes previously reported as fish pathogen. At the top right, genomic features are plotted, including the number of virulence factors (VFs), antibiotic resistance genes (ARs), total genes, the percentage of carbohydrates, protein, ‘Virulence, Disease and Defense’ within the SEED category, GC content and genome length.

In the pan‐genomic analysis using anvi'o workflow (v.7.1), a total of 53,481 genes were identified in 13 *Flavobacteium* genomes. These genes consisted of 15,318 (28.64%) in core, 29,664 (55.47%) in shell (accessory), and 8499 (15.89%) in cloud (unique) (Figure 3). Of these, RSG‐18 genomes predicted a total of 4844 genes, with 1184 cores (24.44%), 3155 shells (65.13%), and 505 clouds (10.43%). Specific genes shared by all genomes included those related to phages, integrases and T1SS pathway that contribute to virulence‐related metabolic pathways. The cloud genes of RSG‐18 included genes encoding phage‐related proteins, such as *GepA*, as well as genes involved in mobilome (prophages, transposons) such as DNA primases and phage‐ or plasmid‐related genes. Additionally, RSG‐18 harbors site‐specific recombinases, adenine‐specific DNA methylase and DNA primases (designed to category L of COGs).

This study offers valuable insights into the potential pathogenicity of *F. plurextorum*, contributing to the prevention and management of fish diseases in the fisheries and aquaculture industries. This study holds significant importance as the first complete genome sequence report on *F. plurextorum* and could lead to more in‐depth research in the future as multiple strains accumulate. For example, a study of the multilocus sequence typing (MLST) system, which is based on nucleic acid polymorphism in genes, can be used to classify strains and investigate evolution of bacteria (Nicolas et al., 2008). Unfortunately, the health status of *S. schlegelii* was not documented at the time RSG‐18 was isolated. However, the RSG‐18 genome showed significant similarity to the type strain of *F. plurextorum* (GCF_002217395) which was isolated from rainbow trout (*O. mykiss*) with bacterial septicemia. To make accurate assessments of potential pathogenicity, extended experimental validation based on these in silico findings is necessary. This can provide insights into the host‐pathogen interactions and their association with environmental conditions.

## AUTHOR CONTRIBUTIONS

**Jisol Lee:** Formal analysis (lead); investigation (lead); methodology (lead); software (lead); writing – original draft (lead); writing – review and editing (equal). **In‐Tae Cha:** Resources (lead); writing – review and editing (equal). **Ki‐Eun Lee:** Resources (equal); writing – review and editing (equal). **Youn Kyoung Son:** Resources (equal); writing – review and editing (equal). **Seoae Cho:** Funding acquisition (lead); project administration (equal); writing – review and editing (supporting). **Donghyeok Seol:** Conceptualization (lead); investigation (equal); supervision (lead); validation (equal); writing – original draft (equal); writing – review and editing (lead).

## CONFLICT OF INTEREST STATEMENT

The authors declare no conflicts of interest.

## Supporting information


**Table S1.** The bioinformatics tools, version and specific parameters used in this study.
**Table S2.** EggNOG functional categories for the predicted genes of RSG‐18.
**Table S3.** 16S rRNA gene sequence identity between RSG‐18 and related strains.
**Table S4.** Amino acid substitutions in *gyrA* between reported antibiotic resistant strains and *Flavobacterium* strains.
**Figure S1.** Colony morphotypes of RSG‐18.
**Figure S2.** Phylogenetic and taxonomic analysis of RSG‐18.
**Figure S3.** SEED subsystem categories of 13 *Flavobacterium* genomes.
**Figure S4.** Subcategories within the ‘Virulence, Disease and Defense’ SEED subsystem of 13 *Flavobacterium* genomes.

**Table S5.** ABRicate results for CARD‐based antibiotic resistance genes and VFDB‐based putative virulence factors.

## Data Availability

The complete genome sequence of *Flavobacterium plurextorum* RSG‐18 has been deposited in NCBI under the accession number GCF_024638035.1.
