# Supplementary material for: Complete genome sequence and potential pathogenic assessment of Flavobacterium plurextorum RSG‐18 isolated from the gut of Schlegel's black rockfish, Sebastes schlegelii
Source: Environ Microbiol Rep. 2024 Jan 31;16(1):e13226. doi: 10.1111/1758-2229.13226 (PMC10878011; doi:10.1111/1758-2229.13226)
Supplement: Supplementary file 1 — Table S1. The bioinformatics tools, version and specific parameters used in this study. Table S2. EggNOG functional categories for the predicted genes of RSG‐18. Table S3. 16S rRNA gene sequence identity between RSG‐18 and related strains. Table S4. Amino acid substitutions in gyrA between reported antibiotic resistant strains and Flavobacterium strains. Figure S1. Colony morphotypes of RSG‐18. Figure S2. Phylogenetic and taxonomic analysis of RSG‐18. Figure S3. SEED subsystem categories of 13 Flavobacterium genomes. Figure S4. Subcategories within the ‘Virulence, Disease and Defense’ SEED subsystem of 13 Flavobacterium genomes. [file EMI4-16-e13226-s002.docx]

**Complete genome sequence and potential pathogenic assessment of *Flavobacterium plurextorum* RSG-18 isolated from the gut of
Schlegel's black rockfish, *Sebastes schlegelii***

Jisol Lee^1^, In-Tae Cha^2^, Ki-Eun Lee^2^, Youn Kyoung Son^2^, Seoae Cho^3*^, and Donghyeok Seol^1,4*^

^1^Department of Agricultural Biotechnology and Research Institute of Agriculture and Life Sciences, Seoul National University, Seoul, South Korea

^2^Microorganism Resources Division, National Institute of Biological Resources, Incheon, South Korea

^3^eGnome, Inc., Seoul, South Korea

^4^Department of Surgery, Seoul National University Bundang Hospital, Seongnam, South Korea

*Correspondence

Seoae Cho, eGnome, Inc., 26 Beobwon‐ro 9‐gil, Songpa‐gu, Seoul 05836, South Korea.

Email: [seoae@egnome.co.kr](mailto:seoae@egnome.co.kr)

Donghyeok Seol, Department of Agricultural Biotechnology and Research Institute of Agriculture and Life Sciences, Seoul National University, 1 Gwanak‐ro, Gwanak‐gu, Seoul 08826, South Korea.

Email: [sdh1621@snu.ac.kr](mailto:sdh1621@snu.ac.kr)

**TABLE OF CONTENTS**

**Supplementary Tables**

**Table S1.** The bioinformatics tools, version and specific parameters used in this study.

**Table S2.** EggNOG functional categories for the predicted genes of RSG-18.

**Table S3.** 16S rRNA gene sequence identity between RSG-18 and related strains.

**Table S4.** Amino acid substitutions in *gyrA* between reported antibiotic resistant strains and *Flavobacterium* strains.

**Table S5.** ABRicate results for CARD-based antibiotic resistance genes and VFDB-based putative virulence factors.

**Supplementary Figures**

**Fig. S1.** Colony morphotypes of RSG-18.

**Fig. S2.** Phylogenetic and taxonomic analysis of RSG-18.

**Fig. S3.** SEED subsystem categories of 13 *Flavobacterium* genomes.

**Fig. S4.** Subcategories within the 'Virulence, Disease and Defense' SEED subsystem of 13 *Flavobacterium* genomes.

| **Objective** | **Program** | **Version** | **Specific parameters used** | **Reference** |
| --- | --- | --- | --- | --- |
| Bam to fastq | Samtools | 1.13 | -fastq (no quality in fastq) | (Danecek et al., 2021) |
| Quality control (QC) | NanoPlot | 1.38.0 |  | (De Coster et al., 2018) |
| *De novo* assembly | Flye | 2.8.3 | -pacbio-raw  -g 4m  --asm-coverage 100 | (Kolmogorov et al., 2019) |
| Polishing | pbmm2 | 1.4.0 |  | a |
|  | GCpp | 2.0.2 |  | a |
| Fixstart | Circlator | 1.5.5 |  | (Hunt et al., 2015) |
| Assessment | BUSCO | 5.2.2 | -l flavobacteriales_odb10 -m genome | (Manni et al., 2021) |
| Annotation | Prokka | 1.14.6 | --rfam | (Seemann, 2014) |
|  | eggNOG-mapper | 2.1.6 |  | (Cantalapiedra et al., 2021) |
|  | RAST | 2.0 |  | (Aziz et al., 2008) |
| Taxonomic analysis | Barrnap | 0.9 |  | b |
|  | BLASTN | 2.13.0 |  | (Boratyn et al., 2013) |
|  | SINA aligner | 1.2.11 |  | (Pruesse et al., 2012) |
|  | trimAl | 1.4.rev15 | -gappyout | (Capella-Gutiérrez et al., 2009) |
|  | IQ-TREE | 2.1.4 | -B 1000 | (Minh et al., 2020) |
|  | Pyani | 0.2.11 |  | (Pritchard et al., 2016) |
| Genome analysis | ABRicate | 1.0.1 | --db CARD  --db VFDB | c |
|  | PHASTER | - |  | (Arndt et al., 2016) |
|  | TXSScan | 1.0.5 |  | (Abby et al., 2016) |
|  | Anvi’o | 7.1 |  | (Eren et al., 2015) |

**Supplementary Tables**

**Table S1.** The bioinformatics tools, version and specific parameters used in this study

^a^ https://github.com/PacificBiosciences

^b^ https://github.com/tseemann/barrnap

^c^ https://github.com/tseemann/abricate

| **COG category** | **Count** |
| --- | --- |
| **Cellular processes and signaling** |  |
| [D] Cell cycle control, cell division, chromosome partitioning | 30 |
| [M] Cell wall/membrane/envelope biogenesis | 350 |
| [N] Cell motility | 14 |
| [O] Post-translational modification, protein turnover and chaperones | 157 |
| [T] Signal transduction mechanisms | 193 |
| [U] Intracellular trafficking, secretion and vesicular transport | 88 |
| [V] Defense mechanisms | 74 |
| [Z] Cytoskeleton | 1 |
| **Information storage and processing** |  |
| [A] RNA processing and modification | 2 |
| [J] Translation, ribosomal structure and biogenesis | 190 |
| [K] Transcription | 346 |
| [L] Replication, recombination and repair | 238 |
| **Metabolism** |  |
| [C] Energy production and conversion | 206 |
| [E] Amino acid transport and metabolism | 297 |
| [F] Nucleotide transport and metabolism | 88 |
| [G] Carbohydrate transport and metabolism | 290 |
| [H] Coenzyme transport and metabolism | 168 |
| [I] Lipid transport and metabolism | 141 |
| [P] Inorganic ion transport and metabolism | 250 |
| [Q] Secondary metabolites biosynthesis, transport and catabolism | 75 |
| **Poorly characterized** |  |
| [S] Function unknown | 1007 |

**Table S2.** EggNOG functional categories for the predicted genes of RSG-18

| **Strain** | **Accession** | **Query Cover (%)** | **Identity (%)** |
| --- | --- | --- | --- |
| *Flavobacterium plurextorum* CCUG 60112^T^ | NR_133747 | **100** | **100** |
| *Flavobacterium plurextorum* 424-08 | HE612091 | 97 | 100 |
| *Flavobacterium plurextorum* 986-08 | HE612092 | 97 | 100 |
| *Flavobacterium plurextorum* JM59 | MN758819 | 98 | 99.93 |
| *Flavobacterium plurextorum* 51B-09 | HE612090 | 97 | 99.93 |
| *Flavobacterium* *plurextorum* Ba20_01 | LC592692 | 100 | 99.79 |
| *Flavobacterium* *pectinovorum* E27CS2 | MK474994 | 98 | 98.85 |

**Table S3.** 16S rRNA gene sequence identity between RSG-18 and related strains

| **Reference** | (Declercq et al., 2021) | (Shah et al., 2012) | (Izumi and Aranishi, 2004) | (Izumi and Aranishi, 2004) |
| --- | --- | --- | --- | --- |
| **Amino acid position** | 82 | 82 | 82 | 87 |
| **Antibiotic susceptible strain** | *F. columnare* ATCC 49512 | *F. psychrophilum* ^a^ | *F. psychrophilum* NCIMB1947 | *F. psychrophilum* NCIMB1947 |
| **Antibiotic resistant strain** | *F. columnare* CDI-A | *F. psychrophilum* F-431-2 | *F. psychrophilum* FPC840 | *F. psychrophilum* FKR9801 |
| **Amino acid substitution** | Ser → Ala | Thr → Arg | Thr → Ala | Asp → Tyr |
| **Strain** |  | | | |
| *F. plurextorum* RSG-18 | Thr | | | Asp |
| *F. plurextorum* 2 | Thr | | | Asp |
| *F. plurextorum* CCUG 60112^T^ | Thr | | | Asp |
| *F. araucananum* DSM 24704^T^ | Thr | | | Asp |
| *F. branchiophilum* DSM 24789^T^ | Thr | | | Asp |
| *F. chilense* DSM 24724^T^ | Thr | | | Asp |
| *F. columnare* NBRC 100251^T^ | Ser | | | Asp |
| *F. hydatis* ATCC 29551^T^ | Thr | | | Asp |
| *F. johnsoniae* UW101^T^ | Thr | | | Asp |
| *F. oncorhynchi* CCUG 59446^T^ | Thr | | | Asp |
| *F. psychrophilum* ATCC 49418^T^ | Thr | | | Asp |
| *F. succinicans* DSM 4002^T^ | Thr | | | Asp |
| *F. tructae* ATCC BAA-2541^T^ | Thr | | | Asp |

**Table S4.** Amino acid substitutions in *gyrA* between reported antibiotic resistant strains and *Flavobacterium* strains

Ser: Serine; Ala: Alanine; Thr: Threonine; Arg: Arginine; Asp: Aspartic acid; Tyr: Tyrosine

**^a^** isolated from brown trout and Atlantic salmon without specifying the name of strain

**Supplementary Figures**


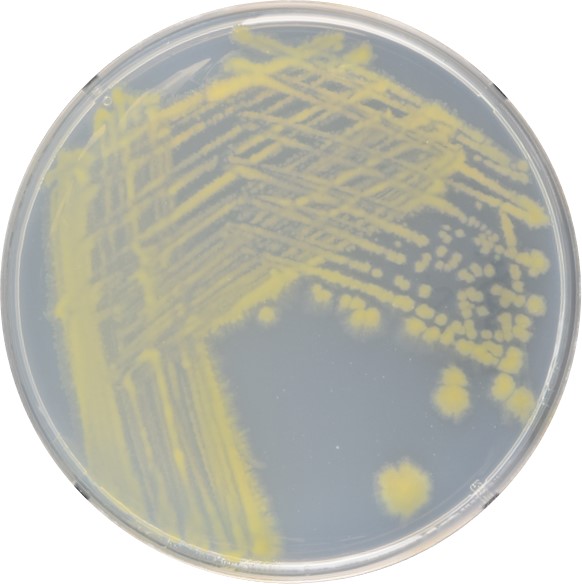


**Figure S1.** Colony morphotypes of RSG-18. A yellow, rough, and irregular colony was observed in strain RSG-18 isolated from the gut of *Sebastes schlegelii* after incubating in an R2A agar at 20°C for 3 days.


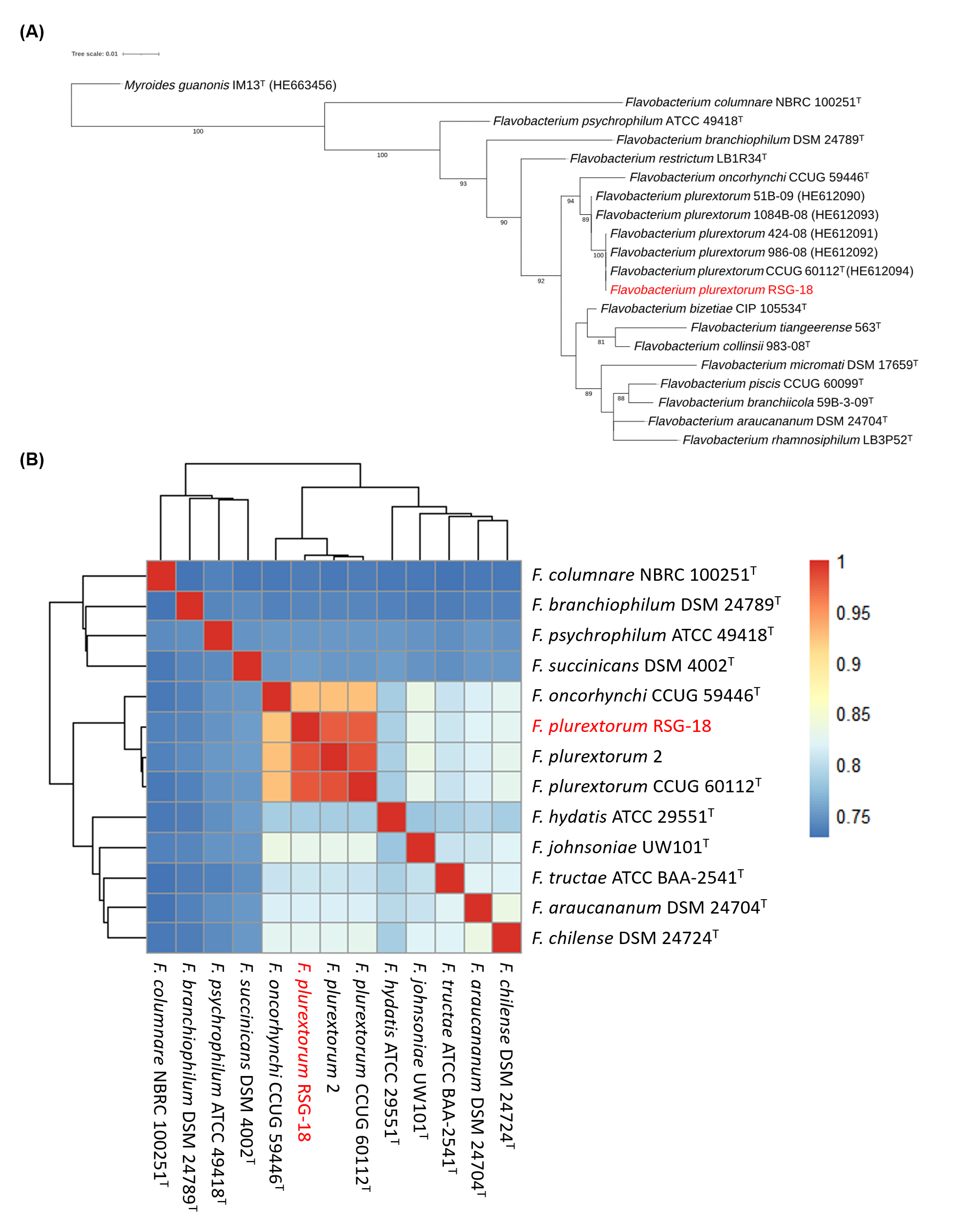


**Figure S2.** Phylogenetic and taxonomic analysis of RSG-18. (A) Phylogenetic tree of the 16S rRNA gene sequences. The phylogenetic tree was constructed based on the 16S rRNA gene sequences of *F. plurextorum* RSG-18 and closely related *Flavobacterium* species. *Myroides guanonis* IM13^T^ was used as an outgroup. Bootstrap values, generated from 1000 replications, are shown for nodes with values greater than 70%. The scale bar represents 0.01 substitutions per nucleotide position. (B) Heatmap of the average nucleotide identity (ANI) values for 13 whole genomes.


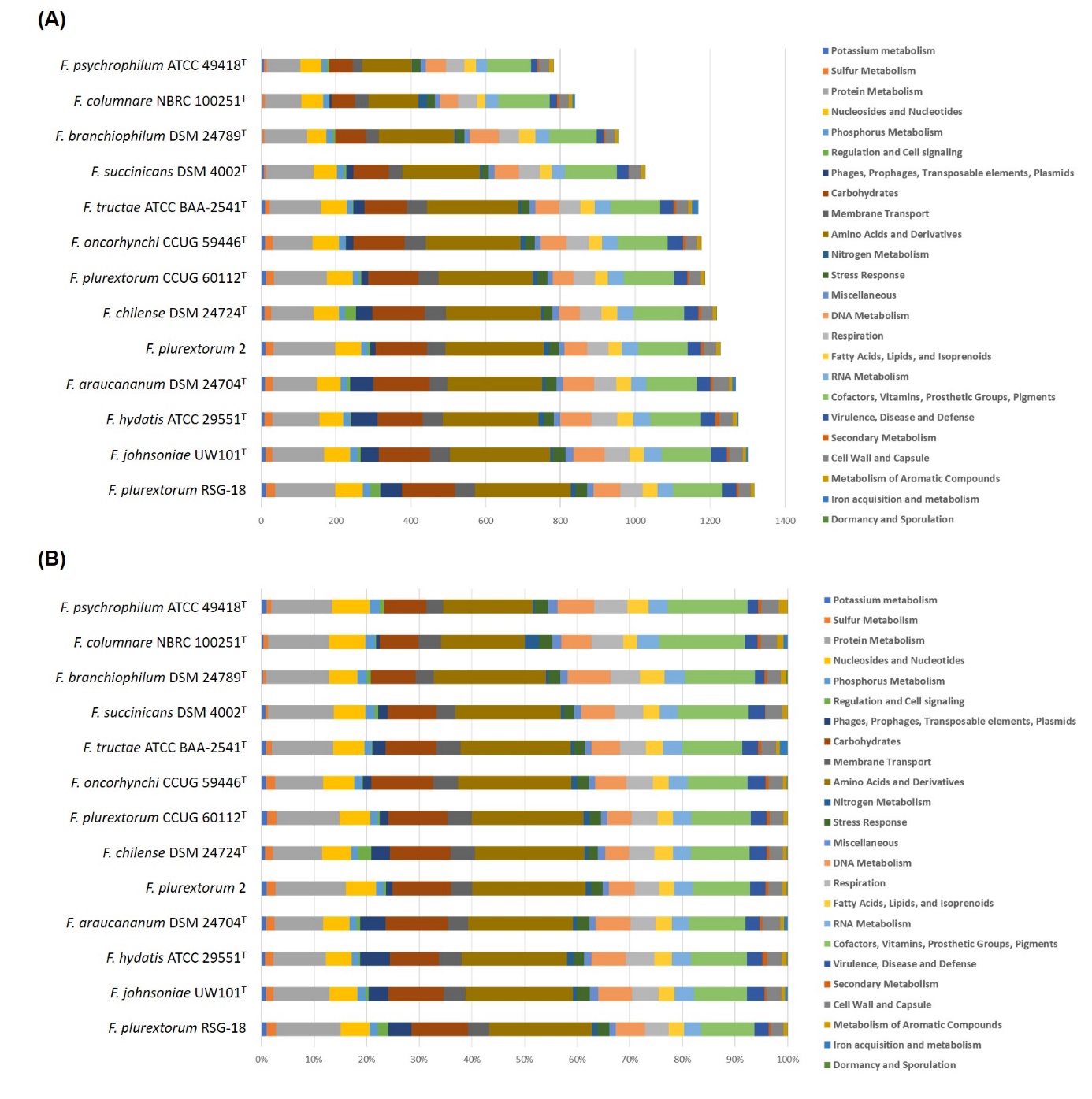


**Figure S3.** SEED subsystem category for *Flavobacterium* genomes. (A) The height of each bar represents the count of genes belonging to that specific category. (B) The 100% stacked bar chart, on the other hand, represents the relative proportions of the SEED subsystem categories within each *Flavobacterium* genome. The length of each segment within the bar represents the proportion of genes belonging to that category in relation to the total gene count for that genome.


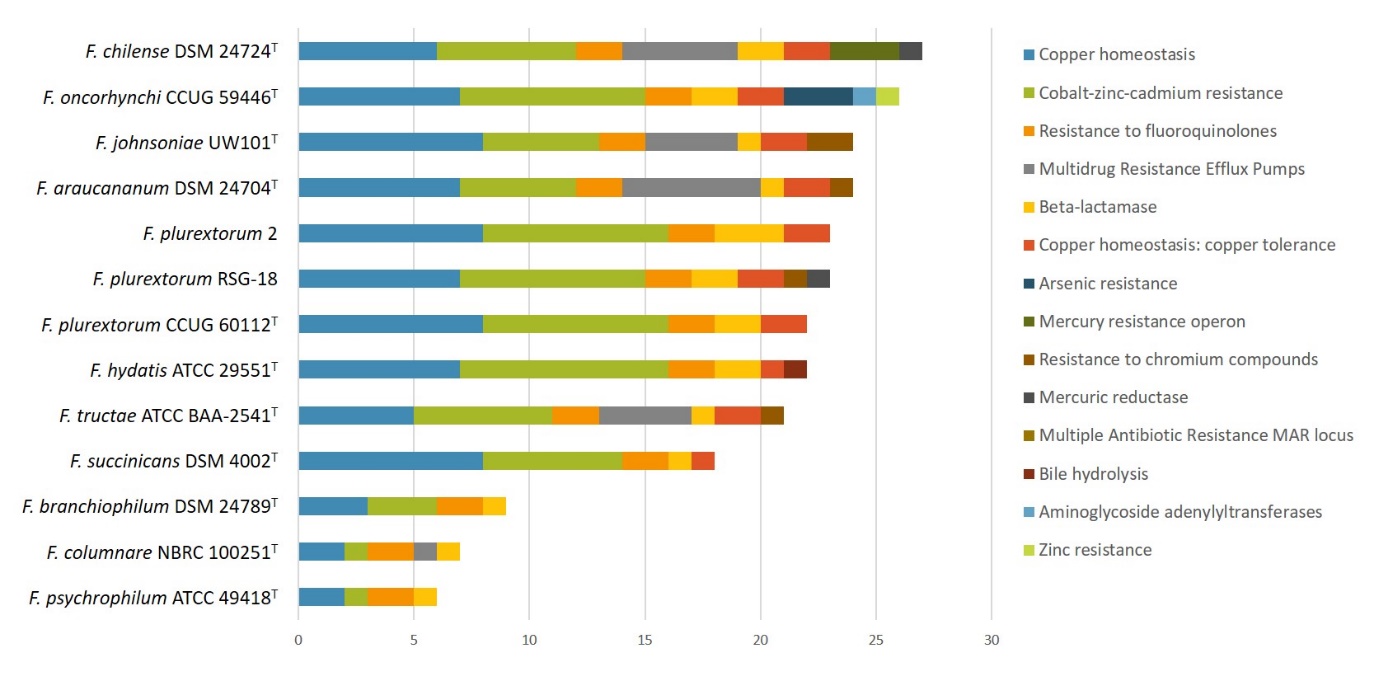
 **Figure S4.** Subcategories within the 'Virulence, Disease and Defense' SEED subsystem for *Flavobacterium* genomes. The height of each bar represents the count of genes belonging to that specific subcategory.

Abby, S.S., Cury, J., Guglielmini, J., Néron, B., Touchon, M., and Rocha, E.P. (2016) Identification of protein secretion systems in bacterial genomes. *Scientific reports* **6**: 1-14.

Arndt, D., Grant, J.R., Marcu, A., Sajed, T., Pon, A., Liang, Y., and Wishart, D.S. (2016) PHASTER: a better, faster version of the PHAST phage search tool. *Nucleic acids research* **44**: W16-W21.

Aziz, R.K., Bartels, D., Best, A.A., DeJongh, M., Disz, T., Edwards, R.A. et al. (2008) The RAST Server: Rapid Annotations using Subsystems Technology. *BMC Genomics* **9**: 75.

Boratyn, G.M., Camacho, C., Cooper, P.S., Coulouris, G., Fong, A., Ma, N. et al. (2013) BLAST: a more efficient report with usability improvements. *Nucleic Acids Research* **41**: W29-W33.

Cantalapiedra, C.P., Hernández-Plaza, A., Letunic, I., Bork, P., and Huerta-Cepas, J. (2021) eggNOG-mapper v2: Functional Annotation, Orthology Assignments, and Domain Prediction at the Metagenomic Scale. *Molecular Biology and Evolution* **38**: 5825-5829.

Capella-Gutiérrez, S., Silla-Martínez, J.M., and Gabaldón, T. (2009) trimAl: a tool for automated alignment trimming in large-scale phylogenetic analyses. *Bioinformatics* **25**: 1972-1973.

Danecek, P., Bonfield, J.K., Liddle, J., Marshall, J., Ohan, V., Pollard, M.O. et al. (2021) Twelve years of SAMtools and BCFtools. *Gigascience* **10**: giab008.

De Coster, W., D’Hert, S., Schultz, D.T., Cruts, M., and Van Broeckhoven, C. (2018) NanoPack: visualizing and processing long-read sequencing data. *Bioinformatics* **34**: 2666-2669.

Declercq, A.M., Tilleman, L., Gansemans, Y., De Witte, C., Haesebrouck, F., Van Nieuwerburgh, F. et al. (2021) Comparative genomics of Flavobacterium columnare unveils novel insights in virulence and antimicrobial resistance mechanisms. *Veterinary research* **52**: 1-13.

Eren, A.M., Esen, Ö.C., Quince, C., Vineis, J.H., Morrison, H.G., Sogin, M.L., and Delmont, T.O. (2015) Anvi’o: an advanced analysis and visualization platform for ‘omics data. *PeerJ* **3**: e1319.

Hunt, M., De Silva, N., Otto, T.D., Parkhill, J., Keane, J.A., and Harris, S.R. (2015) Circlator: automated circularization of genome assemblies using long sequencing reads. *Genome biology* **16**: 1-10.

Izumi, S., and Aranishi, F. (2004) Relationship between gyrA mutations and quinolone resistance in Flavobacterium psychrophilum isolates. *Applied and Environmental Microbiology* **70**: 3968-3972.

Kolmogorov, M., Yuan, J., Lin, Y., and Pevzner, P.A. (2019) Assembly of long, error-prone reads using repeat graphs. *Nature biotechnology* **37**: 540-546.

Manni, M., Berkeley, M.R., Seppey, M., and Zdobnov, E.M. (2021) BUSCO: Assessing Genomic Data Quality and Beyond. *Current Protocols* **1**: e323.

Minh, B.Q., Schmidt, H.A., Chernomor, O., Schrempf, D., Woodhams, M.D., von Haeseler, A., and Lanfear, R. (2020) IQ-TREE 2: New Models and Efficient Methods for Phylogenetic Inference in the Genomic Era. *Molecular Biology and Evolution* **37**: 1530-1534.

Pritchard, L., Glover, R.H., Humphris, S., Elphinstone, J.G., and Toth, I.K. (2016) Genomics and taxonomy in diagnostics for food security: soft-rotting enterobacterial plant pathogens. *Analytical Methods* **8**: 12-24.

Pruesse, E., Peplies, J., and Glöckner, F.O. (2012) SINA: Accurate high-throughput multiple sequence alignment of ribosomal RNA genes. *Bioinformatics* **28**: 1823-1829.

Seemann, T. (2014) Prokka: rapid prokaryotic genome annotation. *Bioinformatics* **30**: 2068-2069.

Shah, S.Q., Nilsen, H., Bottolfsen, K., Colquhoun, D.J., and Sørum, H. (2012) DNA gyrase and topoisomerase IV mutations in quinolone-resistant Flavobacterium psychrophilum isolated from diseased salmonids in Norway. *Microbial Drug Resistance* **18**: 207-214.

**REFERENCES**
